# Women and University Life

**Published:** 1903-05-23

**Authors:** 


### Women and University Life.

An interesting and critical article bearing this title appears in the *Journal of Education* for this month over the signature of Esther Longhurst. It boldly asks what has been the result of opening our universities to women during the last quarter of a century. Has the experiment realised the anticipations of those who were its earnest advocates in the early seventies of last century? Has the peculiar charm of femininity suffered by contact with the higher learning? Is the University woman a cultured woman? Is she more womanly or less after three years of 'Varsity life? The writer of the article, fresh from a discussion on this question at the Edinburgh Conference of Women Workers, concludes without hesitation "that a woman does not gain culture from her stay at a university," and indeed "that the course of study and way of life have a distinctly narrowing effect on her mind." If this be true and this judgment on the sex, by one of them, is to be taken as the final word upon the subject it is a melancholy end to the hopes and aspiration with which the advent of the Girton girl was hailed five and twenty years ago We are inclined to think the judgment is somewhat too general, though we are bound to admit that there is a good deal of truth in some of the contentions which are put forward. It is the experience of most reformers who have lived to see the full adoption of the reforms they advocated that things have not worked out in the way they anticipated when they started on their crusade. It is so with the higher education of women. We do not wish to be misunderstood: we cordially support the opening of the doors of our universities to women; we wish that all universities would imitate the liberal example of the University of London and give with equal hand the same privileges, distinctions, and rewards to women as they do to men. In scores of instances access to university study has been the opening up of intellectual life to women otherwise doomed to fret under the restrictions of narrow domesticity. In medicine there are to-day hundreds of women living full lives of active helpfulness and professional service. The late Sir William Jenner once said he would rather follow his daughter to the grave than to the dissecting-room. Things have changed since then, and the London School of Medicine for women is an outward and visible sign of the triumph of the reformers over the narrow school of thought which Jenner championed in his day. But we must not be blinded to the shortcomings and deficiencies—the unlooked-for bye-products—which experience has brought to light in the evolution of higher education for women.

No doubt much of the diversity of effect which University life produces on different types of the girl undergraduates is in part, if not entirely, due to individual peculiarities in character and temperament. A mode of life which may be fraught with the best possible results for the ambitious non-domestic girl is quite likely to be most unsuitable for the girl whose tastes and affections do not habitually soar far beyond the four walls of her own home.

We can recall more than one instance in which the University girl has in after life seemed to live a less full, a less joyous, and a less womanly life than the Cinderella who stayed at home or the sister who became a hospital nurse. Has not the higher education of women been hitherto on a plan too obviously mannish, too slavishly adopted from the male curriculum to evoke the best and fullest developments of the woman, physical, mental and spiritual? Even when women have followed some branch of natural science for which their industry, patience and conscientiousness have given them special aptitude, how often do they become groovey and narrow, or captivated by some hero-worship become the hewers of wood and drawers of water to some man-master? Their power of acquisition of knowledge is prodigious, but the capacity for assimilation scarcely keeps pace with it, and originality of treatment is rarer than might have been expected. Three years at a University and three years at a hospital are powerfully influential in determining the after life and character of any woman. For imparting *savoir faire*, womanliness in the full and best sense, and even culture, we are not sure that our Minervas must not yield the palm to the hospital nurse.

